# Production of Proliferation- and Differentiation-Competent Porcine Myoblasts for Preclinical Studies in a Porcine Large Animal Model of Muscular Insufficiency

**DOI:** 10.3390/life14020212

**Published:** 2024-01-31

**Authors:** Jasmin Knoll, Bastian Amend, Tanja Abruzzese, Niklas Harland, Arnulf Stenzl, Wilhelm K. Aicher

**Affiliations:** 1 Centre of Medical Research, Department of Urology at UKT, Eberhard-Karls-University, 72072 Tuebingen, Germany; 2 Department of Urology, University of Tuebingen Hospital, 72076 Tuebingen, Germany; bastian.amend@med.uni-tuebingen.de (B.A.);

**Keywords:** cell-based muscle regeneration, large animal model of cell therapy, porcine myoblast, myogenic differentiation, myotubes

## Abstract

Muscular insufficiency is observed in many conditions after injury, chronic inflammation, and especially in elderly populations. Causative cell therapies for muscle deficiencies are not state of the art. Animal models to study the therapy efficacy are, therefore, needed. We developed an improved protocol to produce myoblasts suitable for pre-clinical muscle therapy studies in a large animal model. Myoblasts were isolated from the striated muscle, expanded by employing five different protocols, and characterized on transcript and protein expression levels to determine procedures that yielded optimized regeneration-competent myoblasts and multi-nucleated myotubes. We report that swine skeletal myoblasts proliferated well under improved conditions without signs of cellular senescence, and expressed significant levels of myogenic markers including Pax7, MyoD1, Myf5, MyoG, Des, Myf6, CD56 (*p* ≤ 0.05 each). Upon terminal differentiation, myoblasts ceased proliferation and generated multi-nucleated myotubes. Injection of such myoblasts into the urethral sphincter complex of pigs with sphincter muscle insufficiency yielded an enhanced functional regeneration of this muscle (81.54% of initial level) when compared to the spontaneous regeneration in the sham controls without myoblast injection (67.03% of initial level). We conclude that the optimized production of porcine myoblasts yields cells that seem suitable for preclinical studies of cell therapy in a porcine large animal model of muscle insufficiency.

## 1. Introduction

Severe injury or inflammation of striated muscles may cause permanent muscular weakness and significant impairments in quality of life. Among other disabilities, stress urinary incontinence (SUI) is a major example of a lasting condition caused by muscular deficiency. SUI is a debilitating condition affecting more than 15% of elderly people in our communities [1]. It is associated with psychological distress and may cause social isolation [2]. Moreover, SUI is a challenge to medical personnel and healthcare systems [3]. In women, SUI is associated with pregnancy, vaginal delivery, and menopause [4], and in men, it is a sequela of prostate surgery [5]. For both sexes, injury or persisting inflammation of urogenital structures in the lower pelvic region, neuronal failure, age, and dementia also contribute to SUI. The SUI therapy, therefore, aims at strengthening this muscle complex or supporting its function using other means. For improvement of mild forms of insufficient sphincter muscle function, physical exercise of the lower pelvic floor, complemented by electrophysiological stimulation therapy was shown to improve the muscle strength and thus ameliorate or even cure SUI [6]. Non-surgical treatment of SUI includes also behavioral therapy measures and drug therapy approaches [7,8]. If such a regimen fails to satisfy the patient’s needs, surgical treatment comes into play. Here, options for female and male patients differ considerably. However, for both sexes, injections of bulking agents, the application of sex-adapted tapes or slings, as well as artificial sphincters were used [8]. Yet, such therapies inherit perioperative and also long-term risks and do not treat the underlying conditions. One key mechanism contributing to the development of SUI is the loss of functional muscle tissue or a deficiency of muscle contraction in the urethral closure complex. This motivated research for minimally invasive cell-based SUI therapies to facilitate muscle regeneration [9,10]. Myoblasts are candidate cells for the regeneration of a deficient urethral sphincter complex [10,11] However, despite intensive research, especially in the last two decades, standard cell therapy for SUI of human patients has not been established yet [12,13]. Several preclinical and clinical studies employed myogenic progenitor cells and myoblasts to replace lost or non-functional cells of the striated rhabdosphincter muscle [10] and other striated muscles [14,15].

To explore the regenerative potential of myoblasts for future preclinical studies of cell therapies for muscle deficiency or injury in a surgically relevant large animal model [16], we investigated the cell proliferation, marker gene expression, and differentiation potential of swine striated muscle-derived myoblasts based on two distinct protocols [17,18] and variants of them employing a total of five study cohorts. In a small preclinical feasibility study, myoblasts produced under optimized conditions were injected in a pig model of sphincter muscle insufficiency, and the sphincter regeneration was monitored.

## 2. Materials and Methods

### 2.1. Isolation of Swine Muscle-Derived Cells and Cell Culture Procedures

**Figure 1:**
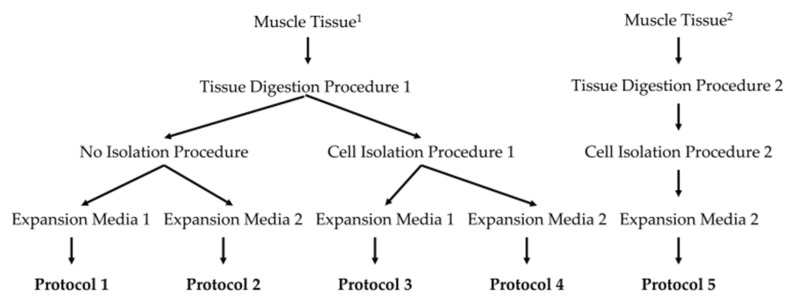
Schematic illustration of the five protocols employed. The different combinations of tissue digestion, cell isolation procedures, and myoblast expansions in two different media according to Metzger et al. [17] or Ding et al. [18], respectively, are summarized. In column 2, footnote ^1^ indicates that the tissue was transported to the lab in PBS-D (144 mM NaCl, 25 mM glucose, 5.4 mM KCl, 14 mM sucrose, 5 mM Na_2_HPO_4_, 1% mL P/S; titrated with NaOH to pH 7.4) complemented with 10% P/S and 10% amphotericin B [17]. Footnote ^2^ indicates that the muscle was transported to the lab in DMEM-HG [18]. In columns 3–5, the number “1” refers to Metzger et al. [17], and the number “2” points to Ding et al. [18].

For the isolation of cells, the *Musculus (M.) semitendinosus* of three fresh cadavers of five to seven-day-old Landrace boars was conducted. Young boars were selected, as their muscles contain more myogenic progenitor cells when compared to muscle samples from older pigs. Tissue samples were excised aseptically, covered with transport media, and brought in sterile containers on wet ice within one hour to the laboratory. Myoblasts were isolated, enriched, and expanded following five distinct procedures based on two different publications [17,18] (Figure 1). The phenotypic appearance of the cells was recorded using microscopy. To determine the duplication rate (DR), cells were washed with cold PBS, detached with the aid of trypsin-EDTA, resuspended in media, counted using trypan blue exclusion in a hematocytometer, and seeded in an inoculation density of 2 × 10^5^ cells per flask. The cells were expanded to approximately 70% confluence, harvested, counted, and expanded again for three consecutive passages in either M-medium [17] or D-medium [18]. Terminal differentiation was induced by incubation of the myoblasts in differentiation media complemented by 2% horse serum as described [18]. Progress of differentiation and generation of syncytia or extended fiber-like cells was recorded using microscopy. The study was approved by the Baden-Wuerttemberg State Authorities under file number CU01-20G.

### 2.2. Evaluation of Gene Expression on Transcript Levels

For RNA extraction, cell expansion media was aspirated, adherent myoblasts were washed twice with PBS, and cells were detached with trypsin-EDTA. Trypsin activity was stopped with the addition of corresponding media supplemented with FBS. The cells were sedimented using centrifugation (700× *g*, 10 min, 4 °C), washed twice with cold PBS, and lysed to extract total RNA with a kit (RNeasy; Qiagen). For cDNA synthesis, the yield and purity of RNA were measured using UV spectrophotometry (Nanodrop; Implen), and 1 μg of RNA was reverse-transcribed using oligo-(dT) primers and MMLV reverse transcriptase (RT) (PrimeScript cDNA synthesis kit; TaKaRa). A quantitative real-time polymerase chain reaction (qPCR) was performed from cDNA using target gene-specific oligonucleotides (Table 1), as described recently [19]. After a hot start (5 min, 95 °C), cDNA was amplified with PCR in 39 cycles (10 s. 95 °C melting, 20 s. 60 °C (or 62 °C for primers of Des and MyoD1) annealing, 30 s. 72 °C extension), and followed by a melting curve analysis for quality management (LightCycler 480 II; Roche). The size of PCR products was confirmed using DNA agarose gel electrophoresis. Expression of swine glyceraldehyde phosphate dehydrogenase (GAPDH) and β2-microglobulin (b2MG) served as housekeeping controls in each run to normalize the transcript levels of the target genes using an efficiency-corrected advanced relative quantification program of the LightCycler480 II as suggested by the instructions for use from the manufacturer [20].

### 2.3. Protein Detection by Immunofluorescence

Myoblasts were grown on chamber slides to the confluence intended. The medium was aspirated; cells were washed twice with cold PBS, fixed with 4% paraformaldehyde (Morphisto, Offenbach, Germany), and permeabilized with 0.1% Triton X-100 (Merck Millipore, Burlington, MA, USA) at ambient temperature. To reduce an unspecific binding of antibodies, samples were preincubated with a blocking solution (5% dry milk powder in 0.1% Tween-20 in PBS (T-PBS)). The blocking solution was aspirated, and the primary anti-desmin antibody (1:200 in 0.1%BSA in PBS; Abcam, Cambridge, UK) was resuspended and incubated in black humidified chambers at 37 °C for 90 min. Unbound primary antibodies were aspirated, and the samples were washed three times with T-PBS. The detection antibody (FITC-F(ab’)_2_-dk-anti-rbt IgG (H+L), 1:200; Jackson ImmunoResearch Laboratories) was added to the samples for incubation in black humidified chambers at 37 °C for 45 min. Samples omitting primary antibodies served as controls. Samples were counterstained with 4′,6-diamidino-2-phenylindole (DAPI; Sigma-Aldrich, St. Louis, MO, USA) to visualize cell nuclei. Staining was visualized and recorded using fluorescence microscopy (Axiovert 200 M, Axiovision software version 4.8; Zeiss, Jena, Germany). Desmin-positive cells were counted with the aid of ImageJ software version 1.53, NIH) in comparison to all cells visualized with DAPI blue nuclei.

### 2.4. Flow Cytometry

Flow cytometry (FC) was employed to explore the expression of myogenic marker CD56 on the myoblasts [26,27]. The myoblasts were detached using mild proteolysis (Accutase; SigmaAldrich, St. Louis, MO, USA), and aliquots of 5 × 10^5^ cells were resuspended in PFEA sample buffer and sedimented in microtubes [26]. To avoid unspecific antibody binding, cells were incubated with pre-immune serum (Gamunex, 1:20 in PBS; 20 min, 0 °C; Grifols SA, Barcelona Spain [26], washed, and incubated at 0 °C for 20 min with a cross-reactive PE-labeled antibody to human CD56 (NCAM, diluted 1:10 in PFEA; BioLegend, San Diego, CA, USA). Samples were diluted with PFEA. The cells were sedimented using centrifugation, and the supernatant with unbound antibodies was discarded. The cells were resuspended in 300 µL PFEA. The size and granularity of the cells were recorded with FC employing forward (FSC-A) and side scatter (SSC-A; LSRII; BD Bioscience, Franklin Lakes, NJ, USA) to select living cells. Then, antibody staining intensities were measured using FC [26,27]. Cells with no antibody staining and FC compensation particles (BD Bioscience) were employed for gating. Data were evaluated using FACS-Diva (version 6.1.3.) and FlowJo software (version 10.10, both BD Bioscience). The FCS- and SSC-scatters are presented as 2D dot blots, median fluorescence intensity (MFI) of cells staining as histograms (thin black line), and background fluorescence of unstained cells as shaded histograms (grey).

### 2.5. Efficacy of Myoblast Therapy in a Preclinical Animal Study

For a small pre-clinical feasibility study, a litter of one additional boar and the corresponding five female landrace pigs were selected. Myoblasts for cell therapy were prepared from the male pig as described above, following Prot.4 (Figure 1). Urethral sphincter muscle deficiency was induced in the female landrace littermates through transurethral electrocautery and balloon dilatation [16]. The sphincter deficiency was confirmed by measuring the urethral closure pressure immediately after the surgery as described (i.e., urodynamics; Aquarius TT, Laborie) [16,28] and normalized in each gilt to the initial closure pressure levels determined before surgery (=100%). The functional deficiency of the urethral closure complex was confirmed during a follow-up in each gilt with urodynamics three days after induction of sphincter muscle injury and before myoblast injections. Then, myoblasts (1 × 10^6^ total in two aliquots of 250 μL each) were injected with a Williams needle (Boston Scientific) in the injured urethral sphincter muscle employing a cystoscope under visual control as described [28]. Functional regeneration of the sphincter complex was determined on day 35 after induction of muscle injury which corresponds to day 32 after cell therapy. Using this strategy, homologous cells from the male littermate will not be rejected by the female littermate recipients upon injection. Consequently, immune suppression is not required which could bias the outcome, especially in feasibility studies with small cohort sizes. But in future studies, the Y-chromosome of male cells could be detected using in situ hybridization or other techniques if needed. Six pigs with induced sphincter deficiency but without cell injections served as the control cohort.

### 2.6. Statistics

Data generated were processed with Excel (Microsoft). Statistical analyses were performed with GraphPad Prism version 5.04 for Windows (GraphPad Software) and IBM SPSS Statistics for Windows, version 29.0 (Armonk: IBM Corpversion 26). Depending on the normality distribution, either normal data were used, or the data were transformed via log10 to result in an approximately normal distribution. Afterwards, one way ANOVAs were performed. The one way ANOVAs were completed using post hoc tests of Tukey. If the transformation to normal distribution failed non-parametric tests were performed (Mann–Whitney U or Kruskal–Wallis Test). *p*-values between 0.05 and 0.01 were considered significant (*), between 0.01 and 0.001 very significant (**), and *p*-values smaller than 0.001 as highly significant (***).

## 3. Results

### 3.1. Proliferation and Morphology of Swine Myoblasts

**Figure 2:**
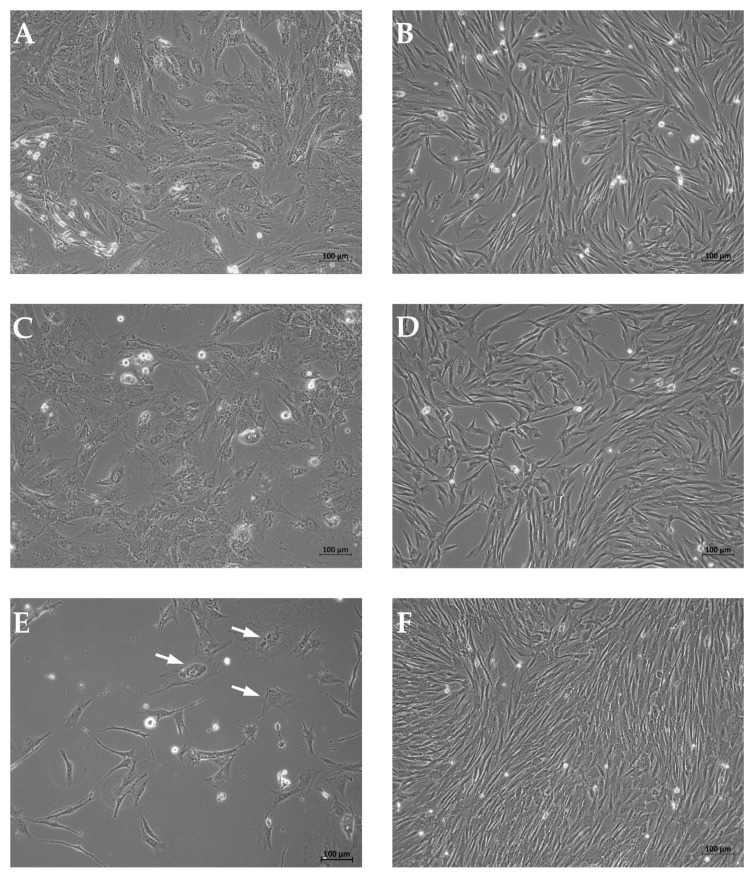
Morphology of myoblasts expanded following different protocols. Myoblasts produced following Prot.1 (**A**) and Prot.3 (**C**) appeared wider and contained more granula when compared to cells expanded following Prot.2 (**B**), Prot.4 (**D**), or Prot.5 (**F**), respectively. Myoblasts expanded in M-media (left panel) contained more cells with a perinuclear hallow or stretched cells ((**E**), arrows). Size bars indicate 100 μm. The pictures are representative artwork for cultures generated from the three individual boars and expanded using different protocols included in this study.

Myoblasts were prepared from three individual boars. The cells were expanded employing five different procedures Prot.1 to Prot.5 (Figure 1), in either M-media containing horse serum [17] or in D-medium complemented with bFGF [18]. The mean cell duplication rate (DR) of cells in M-media containing horse serum was significantly different (mean DR: M-media: 1.99) when compared to cell expansion in D-media (mean DR: D-media: 1.37; *p* = 0.038, Mann–Whitney U Test). Cells produced according to Prot.3 presented with the lowest mitotic activity (DR: 2.21), while cells produced using Prot.4 showed the highest (DR: 1.18). Furthermore, cells appeared with different morphologies (Figure 2). Cells produced with Prot.1 and Prot.3 appeared with a wider somata and increased granularity (Figure 2A,C). Several cells produced using these protocols appeared flat, enlarged, and stretched (Figure 2E). This may resemble features of replicative senescence [29]. Myoblasts produced using Prot.2, Prot.4, and Prot.5 appeared slim and without prominent granularity (Figure 2B,D,F).

### 3.2. Expression of Transcripts Encoding Myogenic Markers

**Figure 3:**
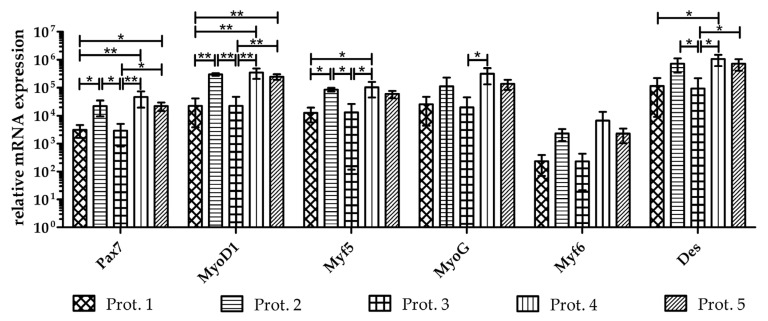
Transcript expression of myogenic marker genes in myoblasts. Expression of myogenic markers was enumerated using RT-qPCR of RNA isolated from cells of all three donors upon expansion to the third passage following protocols as indicated in three individual sets of experiments. Cells expanded in D-media expressed all myogenic markers at higher levels when compared to cells expanded in M-media. The data present the mean values ± standard deviations of target gene expression normalized to housekeeping controls. Significant differences are indicated: one way ANOVAs with Tukey post hoc tests performed on log-transformed values: *, **. All one way ANOVAs calculated displayed significant results (Table 2) even if post hoc tests could not identify the groupwise differences for all genes (e.g., Myf6).

Several marker genes have been identified that play a central role in myogenesis. The expression of the gene paired box 7 (Pax7), which is specific for proliferating myoblasts, and several of the myogenic regulatory factors (MRFs), including myoblast determination protein 1 (MyoD1), myogenic factor 5 (Myf5, myogenin (MyoG), myogenic factor 6 (Myf6, alias MRF4) [30,31], as well as expression levels of the intermediate filament desmin (Des) found in muscle was investigated using RT-qPCR in myoblasts expanded with the five different protocols (Figure 3). Myoblasts generated using Prot.4 expressed all myogenic markers at the highest levels, followed by the cells produced using Prot.2 and Prot.5. Again, myoblasts generated using Prot.1 and Prot.3 significantly lagged behind (Figure 3 and Table 2).

In addition, the expression of skeletal muscle alpha actin 1 (ACTA1), myosin light chain-1 (Myl1), myostatin (MSTN), myosin heavy chain 1 (MYH1), and actin (ACT) were detected using RT-qPCR as well. However, significant differences between cells produced with Prot.1 to Prot.5 were not recorded (data not shown). We conclude that the expansion of porcine myoblasts in D-medium containing bFGF favored a skeletal muscle phenotype of porcine muscle-derived cells.

### 3.3. Detection of CD56 Using Flow Cytometry

**Figure 4:**
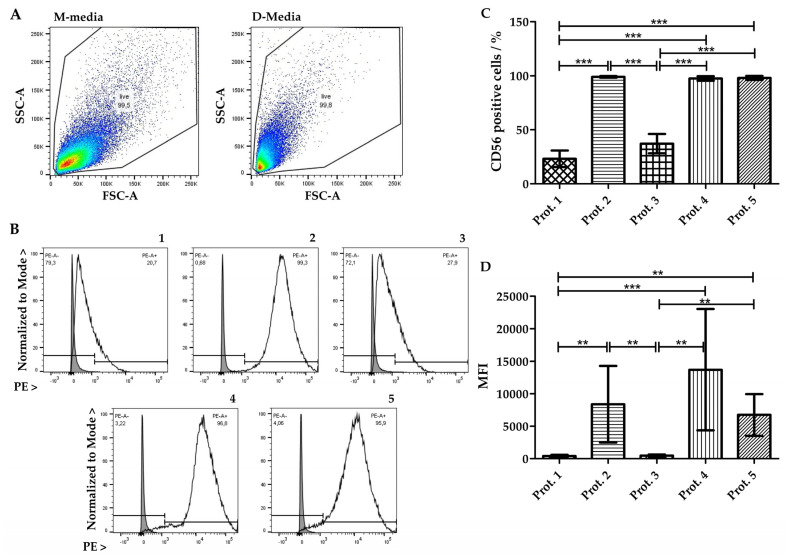
Characterization of myoblasts using flow cytometry (FC). (**A**) The size (forward scatter; FSC-A) and complexity (side scatter; SSC-A) of myoblasts were determined using FC. (**B**) Myoblasts expanded in M-media showed a broader distribution in FSC and SSC, when compared to cells in D-media. The expression of CD56 was enumerated using FC as well. Representative cells derived from the three boars and expanded with procedures following Prot.1 to Prot.5 are displayed as indicated in panels (**B1**–**B5**). Expression of CD56 is higher in cells expanded in D-media (**B2**,**B4**,**B5**), when compared to cells from M-media (**B1**,**B3**), respectively. (**C**) Gating of CD56-negative cells (see (**B**)) facilitated the enumeration of CD56-positive cells. Significant differences were found between cells produced in the different protocols as indicated. One way ANOVA (*p* < 0.001) with Tukey’s post hoc-tests (***). (**D**) The median fluorescence intensities (MFI) (see (**B**)) of the different cells are depicted as well. Cells produced in M-media expressed less CD56 per cell which translated into a low MFI when compared to cells in D-media. Similar significant differences were found between cells produced in the different protocols as indicated: one way ANOVA (*p* < 0.001) with Tukey post hoc tests performed on log-transformed values (**, ***). The data presents the mean values ± standard deviations from three independent experiments with cells from the three donors expanded in the different media.

The cell adhesion molecule CD56 is a multifunctional protein which is expressed in satellite cells and myoblasts [32,33,34]. We therefore investigated expression of CD56 on swine myoblasts by FC in three independent sets of analyses (Figure 4). Differences in cell size and granularity observed by microscopy were confirmed by FC, as myoblasts expanded in M-media had a wider scatter and particle distribution when compared to myoblasts expanded in D-media (Figure 4A). Expression of CD56 in cells expanded in M-media following Prot.1 and Prot.3 was very low (Figure 4B(1,3)). A clear separation of the staining peak above background was barely recorded in these cells. The percentage of CD56 positive myoblasts was raised highly significant in cells expanded in D-media when compared to cells in M-media (Figure 4B(2,4,5),C). Additionally, the means of MFI of myoblasts cultivated in K-media (Prot.1: 410, Prot.3: 475) were lower than the means of MFI of myoblasts cultivated in D-media (Prot.2: 8379, Prot.4: 13700, Prot.5: 6730). The highest expression levels among all five protocols were recorded in Prot.4 cells (Figure 4D). This corroborated that elevated expression of CD56 on porcine myoblasts was facilitated by cell expansion in the D-media lacking horse serum.

### 3.4. Expression of Desmin in Swine Myoblasts

**Figure 5:**
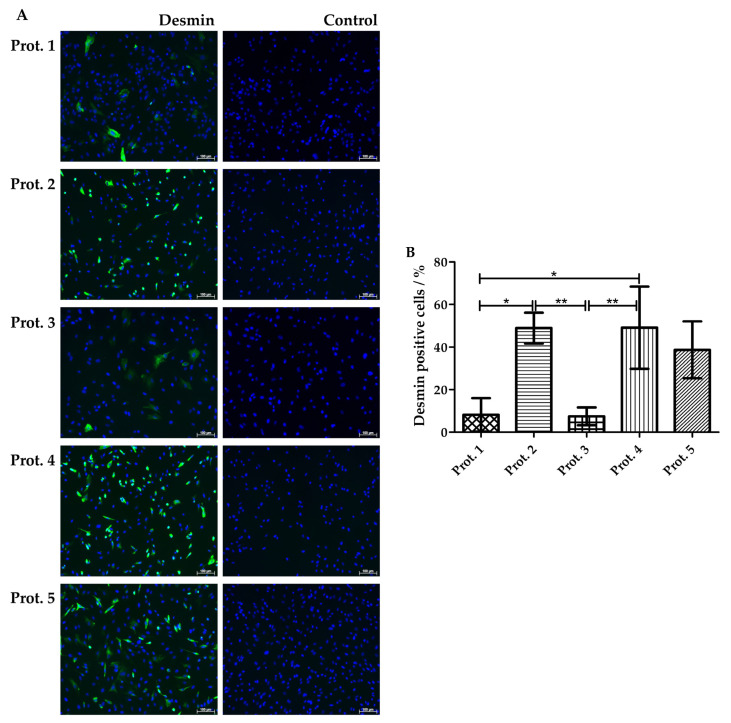
Detection of intracellular desmin using immunofluorescence. (**A**) Myoblasts were prepared following the five different protocols as indicated and fixed, and stained with anti-desmin reagents as indicated (left panel). Incubation of cells with a secondary antibody only served as a control (right panel). Nuclei were visualized with DAPI. The micrographs are representative of cells from all batches included in this study. More cells expressed desmin upon expansion in D-media when compared to cells in M-media. Size bars indicate 100 μm. (**B**) The percentage of desmin-expressing cells as a function of the protocol employed was enumerated in micrographs using ImageJ. Desmin was detected in significantly less myoblasts generated with Prot.1 or Prot.3 when compared to cells produced using Prot.2, Prot.4, or Prot.5. The data present the mean values of three independent experiments ± standard deviations. Significant differences are indicated: on way ANOVA (*p* = 0.002) with Tukey post hoc tests (*, **).

Desmin is a muscle-specific intermediate filament contributing to the proper structure and function of contractile cells [35,36]. We, therefore, compared the expression of desmin in myoblasts from all three individual donors after expansion using the five different protocols employing immunofluorescence (IF). Desmin was detected using IF in all batches generated (Figure 5A). As the expression levels of an individual protein cannot be delineated from IF signal intensities directly, nuclei were counterstained using DAPI, allowing the enumeration of cell counts of desmin-positive versus desmin-negative myoblasts (Figure 5B). Populations produced using Prot.2 (48.9%; *), Prot.4 (49.1%, *), and Prot.5 (38.7%; not significant) presented with significantly more desmin-positive cells, when compared to Prot.1 (8.2%) and Prot.3 (7.4%) cultures (Figure 5B). This confirmed that culture of porcine muscle-derived cells in D-media yielded populations enriched with myoblasts when compared to the same cells expanded in M-media.

### 3.5. Myogenic Differentiation to Generate Myotubes

**Figure 6:**
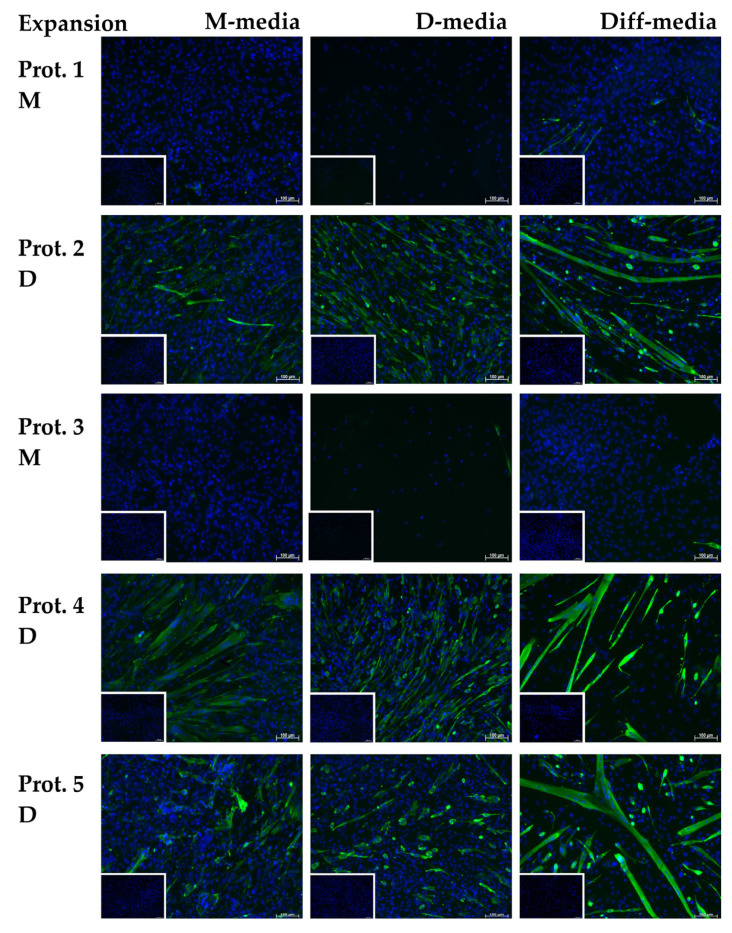
Terminal differentiation of myoblasts. Myoblasts were expanded using Prot.1 and Prot.5 in M- or D-medium and maintained in M- and D-medium for controls as indicated. Differentiation of cells was induced. Generation of elongated muscle fibers was recorded using immunofluorescence of desmin expression. Nuclei were counter-stained with DAPI. Myoblasts expanded in D-media (Prot.2, Prot.4, Prot.5) generated multi-nucleated elongated myotubes, while cells expanded using Prot.1 and Prot.3 died upon changing to D-medium and did not generate myotubes efficiently. Inserts show control staining omitting the primary antibody. Size markers indicate 100 μm. The figure shows representative micrographs from a series of three independent experiment using cells from three individual boars each.

Terminal differentiation and formation of myotubes were stimulated in myoblasts, expanded from cells of all three donor animals using Prot.1 to Prot.5, and differentiated following the established protocols [18]. As controls, cells were maintained in either M-media (Figure 6, left column) or D-media (Figure 6, middle column). After incubation in differentiation media (Figure 6, right column), extension of cells was noted using microscopy in cells produced using Prot.2, Prot.4, and Prot.5 after 2–3 days (not shown). After 4 days of differentiation, cells were fixed to visualize desmin-expressing myoblasts and multinucleated myotubes using IF (Figure 6). Myoblasts expanded following Prot.2, Prot.4, and Prot.5, yielded long myotubes and expressed desmin (Figure 6), while the other cultures yielded short and less (Prot.1) or no (Prot.3) elongated myotubes (Figure 6).

**Figure 7:**
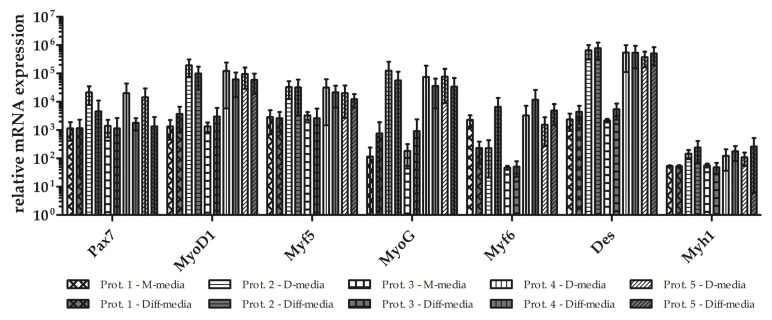
Expression of marker genes in myotubes. Transcripts encoding myogenic marker genes were enumerated using RT-qPCR in Prot.1 to Prot.5 cells after terminal differentiation and in corresponding controls. Data are mean values ± standard differentiation from three independent experiments using cells from three donors produced using the five different protocols. Most overall test values appeared to be significant (Kruskal–Wallis Test) (Table 3) except for Pax7 and Myh1. However, all post tests performed were not significant.

Most of the myoblasts expanded initially in M-media (Prot.1, Prot.3) did not survive the media change to D-media. Only very few intact cells and nuclei were recorded after DAPI staining. Yet, they survived the media change to differentiation media, however, without efficient myotube formation (Figure 6). Interestingly, cells expanded initially in D-media survived the media change to M-media, and even seemed to undergo differentiation as well (Figure 6). This difference in syncytia and myotube formation was corroborated by investigating the transcripts of the differentiated populations (Figure 7 and Table 3). Expression of Pax7 was low in myoblasts and remained low in myotubes of cells generated using Prot.1 and Prot.3.myotubes.

Expression of MyoD1, Myf5, and Des was comparably low, and transcripts encoding Myf6 were reduced in Prot.1 cells or remained low in Prot.3 cells after differentiation (Figure 7). In contrast, the expression of Pax7 was elevated in Prot.2, Prot.4, and Prot.5 myoblasts, and was reduced upon differentiation (Figure 7). Myf6, a factor associated with terminal differentiation, was elevated in myotubes differentiated from Prot.2, Prot.4, and Prot.5 cells, respectively (Figure 7). The changes in transcript levels recorded for Pax7 and Myf6 confirmed the success of terminal differentiation of porcine myoblasts. [37,38]. Expression of Des was and stayed high in Prot.2, Prot.4, and Prot.5 cells (Figure 7). We conclude that the expansion of porcine muscle-derived cells in D-media facilitated the expansion of differentiation-competent myoblasts without promoting premature replicative senescence.

### 3.6. Myoblast Therapy of a Deficient Sphincter Muscle in the Animal Model of Incontinence

**Figure 8:**
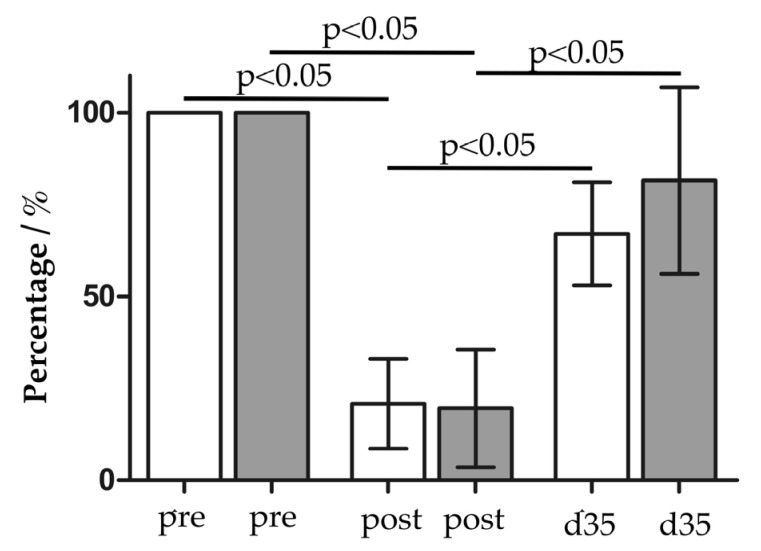
Regeneration of the deficient urethral sphincter muscle with myoblast injection. The urethral muscle strength was determined using urodynamics in each gilt before (pre) and after (post) induction of sphincter muscle deficiency, and muscle deficiency was significant (*p* < 0.05). On day 3 after muscle injury, myoblasts were injected in the urethrae (grey bars, n = 5). Gilts without cell therapy served as controls (white bars, n = 6). On day 35 (d35) after muscle injury, the functional recovery was determined with urodynamics again. Injection of myoblasts facilitated a functional recovery (81.54%, grey bar d35) which was significantly above the muscle strength determined after muscle injury (grey bar post, *p* < 0.05), but not significantly above spontaneous regeneration (white bar d35). Spontaneous regeneration was significant as well (67.03%, white bar d35, *p* < 0.05). The graph presents the mean urodynamics of each cohort ± standard deviation after normalizing the data for each animal to the muscle strength recorded before the muscle injury.

Urinary incontinence was surgically induced in five female pigs through transurethral electrocautery and balloon dilatation [16]. This reduced the mean urethral wall pressure significantly in both cohorts, in the cell therapy group (19.64% ± 15.99%, *p* < 0.05, n = 5) and in the control group (to 20.83% ± 12.21%, *p* < 0.05, n = 6), respectively, when compared to the initial levels (=100%; Figure 8). Three days after induction of sphincter deficiency, 1 × 10^6^ myoblasts, produced by following Prot.4 from *M. semitendinosus* of the male littermate, were injected into the injured urethrae. The regeneration of the sphincter complex was determined using urodynamics thirty two days after myoblast injection, which corresponds to thirty five days after muscle injury (Figure 8). The spontaneous recovery from the induced urethral muscle deficiency w/o myoblast injection was significant, and reached 67.03% ± 14.00% (*p* < 0.05). The injection of Prot.4 myoblasts yielded better functional regeneration and was significant as well (81.54% ± 25.40%, *p* < 0.05) when compared to the urethral wall pressure after induction of sphincter injury. After follow-up, the sphincter regeneration facilitated using myoblast injection was elevated in comparison to the mock-treated controls but not significantly different from spontaneous recovery (Figure 8).

## 4. Discussion

The success of preclinical animal studies paved the way for clinical feasibility studies aiming at cell therapy of stress urinary incontinence (SUI) with autologous cells [9,39,40]. Despite initial enthusiasm some years ago, cell therapy of SUI is not yet a clinical standard [12,13]. Various reasons for this seemingly contradictory situation must be considered, including the lineage and quality of the cells applied in preclinical animal studies. The tissue targeted with SUI cell therapy—the urethral sphincter muscle—consists of parts of smooth muscle tissue, which resembles, anatomically, the lissosphincter [16]. In addition, striated muscle cells of the outer rhabdosphincter reinforce the contractile complex [16]. Therefore, either stromal cells derived, for instance from adipose tissue [41] or skeletal myoblasts [10,11,42] were employed in both, preclinical or clinical studies [9,43]. Production of regeneration-competent human mesenchymal stromal cells from bone marrow, adipose tissue, and other sources as well as the production of skeletal myoblasts, e.g., from *M. soleus* under good medical procedure conditions is state of the art [10,11,42,43]. In contrast, production protocols or phenotypes of cells applied in animal SUI studies were not always disclosed in detail, thus impeding the interpretation of therapy versus outcome [44]. In some preclinical animal studies, the myoblasts employed were investigated before their application to some extent [45,46,47]. However, sufficient information regarding the quality measures of porcine muscle cells, which had been used in SUI models for regenerative therapy, has not been found.

In preclinical and clinical studies, quality measures for muscle cells applied should address several analyses, including phenotype, stage of differentiation, stress resilience, and replicative senescence. Expression of Pax-7, MyoD-1, and Myf-5 are recorded in activated satellite cells and proliferating skeletal myoblasts and, therefore, seem specific for the desired phenotype [30,31,38,48]. Myogenin is especially expressed during the myogenesis [38], whereas desmin, a muscle-specific intermediate filament, is found in different stages of myoblasts’ differentiation corresponding to the prominent expression in our cultures [35]. The expression profile of cells expanded with Prot.4 showed the highest accordance with the expected markers of myoblasts. Moreover, the high expression of the cell surface molecule CD56 on myoblasts was in complete correlation with the elevated expression of all myogenic markers investigated and thus may serve as a tool to enrich regeneration-competent myoblasts.

The source of cells used for cell-based therapy or tissue engineering may also influence the outcome. In clinical situations, *M. soleus* is one of the preferred sources for myoblast production [10]. Even-toed ungulates such as pigs do not have an *M. soleus*. In our preparatory studies, we, therefore, explored the phenotypes of myoblasts isolated from different skeletal muscles of adult animals. Except for desmin, the expression of the markers investigated in cells from adult pigs was not quite convincing (not shown). To achieve the first elements of improved myoblast culture protocols, we, therefore, included in this study only cells isolated from *M. semitendinosus* of very young boars, as they contained sufficient amounts of myogenic progenitors, including satellite cells [49], and we optimized the expansion media. Of note, muscle tissue may contain fast- and slow-twitch fibers. In the omega-shaped rhabdosphincter muscle type I slow-twitch fibers prevail and enable baseline tonic activity and to a large part voluntary sphincter control. Depending on the clinical situation, this difference in muscle type may influence the outcome of cell therapy as well.

The differences in the composition of M- versus D-media merits discussion. The M-media contained high-glucose DMEM supplemented with 10% bovine plus 10% horse serum, L-glutamine and antibiotics [17]. It resulted in slower cell proliferation as determined by the duplication rates of myoblasts. More cells containing granula, showed perinucler hallows, or seemed stretched. This appearance of cell somata was associated with replicative senescence [29]. However, additional experiments in the future must explore whether senescence is facilitated by the expansion of porcine myoblasts in M-media. The D-media contained F10 medium, complemented by 15% FBS, FGF, and antibiotics [18]. Above all, the D-media facilitated cell proliferation and yielded a significant expression of a variety of muscle cell markers. Thus, the expansion of porcine myoblasts in D-media yielded cells with a promising phenotype. Recently, horse serum was used as a stimulus to induce the differentiation of preconditioned mesenchymal stromal cells to become elongated myogenin-positive cells [50], while serum deprivation of C2C12 myoblasts reduced the percentage of myogenin- and MyoD-positive cells, indicating a reduction of cells undergoing terminal differentiation and cell fusion [51]. In our study, the complementation of differentiation media using horse serum facilitated the generation of multinucleated myotubes in cells expanded in D-media. This was not only obvious through comparing the number of elongated myotubes expressing desmin, but also when investigating the changes in marker gene expression after differentiation. The phenotype of proliferation-competent myoblasts and myotubes formation is also regulated in vitro with aFGF (FGF-1) and bFGF (FGF-2) [52]. While autocrine and exogenous FGF supported the expansion of myoblasts and blocked terminal differentiation, deprivation of bFGF from myoblast media facilitated the generation of myotubes [18].

We noted that the cells expanded ex vivo in M-media did not survive a media change to D-media. This indicated that the factors contained in M-media allowed for myoblast expansion but failed to mount resilience to cell death. The cell death observed after a change from M- to D-media was associated with a kind of factor deprivation. Injection of cells in a tissue is known to cause stress [53]. Of note, cell death upon media change correlated with low expression of CD56 in myoblasts expanded in M-media, and CD56 expression is needed for efficient muscle differentiation [33]. CD56 was also associated with resistance to cellular stress [54]. Resilience to cell death after injection may be a key to the success of the clinical application of cells. This, again, suggests that the analysis of CD56 expression could become a part of the quality measures for future myoblast cell therapies.

However, upon isolation of cells from three individual boars from three independent litters and different parents, we noted that myoblasts from the third boar expressed the marker genes investigated at lower levels when compared to the two earlier preparations (not shown). As the cells were produced under identical procedures and protocols, to the best of our knowledge, we conclude that interindividual differences in tissue characteristics may exist in domestic pigs. Such interindividual differences were noted, for instance, using epigenetic analyses of distinct cohorts [55]. These individual disparities in cell characteristics could in part contribute to the partially conflicting data reported from clinical cell therapies [56,57]. Complementing quality measures using epigenetic analyses as well as selection or enrichment of regeneration-competent cells, for instance through sorting for CD56^pos^ myoblasts, may yield better clinical outcomes as well. However, the definition of suitable markers for human myoblast enrichment must await further studies.

In our small exploratory study, injection of homologous myoblasts from a male littermate facilitated a functional regeneration of the injured urethral sphincter complex when compared to mock-treated incontinent pigs that did not receive the cell therapy. A significant difference between spontaneous recovery and myoblast-mediated sphincter regeneration has not been achieved yet. The reasons for these results include the small cohort size of five animals only. Based on the improved proliferation capacities, cell characteristics, and specifically the high CD56 expression, MPCs produced using Prot.4 were selected for the animal study. Moreover, due to the limitations in cohort sizes available for this exploratory study, only one injection dose, i.e., 1 × 10^6^ myoblasts, was explored following our previous studies [28]. Thus, in future studies, larger animal cohorts and dose-response curves must be included. Moreover, all cells included in this study were isolated from *M. semitendinosus*. Harvesting cells from this large muscle is technically simple, and, therefore, *prima vista is* advantageous. As indicated above, in our next studies myoblasts will be isolated from other muscles, expanded according to Prot.4, and characterized to explore their regenerative potential in incontinent pigs. Expression of CD56 on such myoblasts will probably play a central role in these future studies. Upon successful completion, such porcine myoblasts may then be applied to cell therapy studies in large animal models of other muscle deficiencies as well, including, e.g., deficiency of the anus muscle associated with stool incontinence, striated muscles after accidents, injuries, or (tumors) surgeries, and, after adaptation, possibly even for myoblast therapy for heart attacks.

## 5. Conclusions

We conclude that the phenotype of swine myoblasts differs quite considerably depending on the composition of the expansion media employed. Complementing expansion media with 15% FBS and bFGF seems preferable over the addition of horse serum. The phenotype of porcine myoblasts seems to depend to a lesser extent on the technique employed for the isolation of cells from muscle tissue samples but rather on protocols used for cell expansion. For the generation of porcine myoblasts for preclinical animal studies targeting skeletal muscles, we recommend the isolation of cells from young donors, following Protocol 4 for cell expansion, and the enrichment of CD56 expressing myoblasts.

## Figures and Tables

**Table 1:** Oligonucleotides employed for quantitative reverse-transcription polymerase chain reaction of swine cDNAs in 5′-3′ orientation, including gene bank access, reference, and expected DNA product size in base pairs. Of note, the b2MG and ACT primers also amplify alternative transcripts. For b2MG: ^#^ XM_021096362.1, for ACT: ^##^ XM_021071931.1 and XM_021071930.1, respectively.

Gen	Forward Sequence	Reverse Sequence	Accession No.	Reference	Size
GAPDH	CCATCACCATCTTCCAGGAG	ACAGTCTTCTGGGTGGCAGT	NM_001206359.1	[21]}	346
b2MG	ACGGAAAGCCAAATTACCTGAACTG	TCTGTGATGCCGGTTAGTGGTCT	NM_213978.1 ^#^	[22]	261
MyoG	CGCCATCCAGTACATCGAG	TGTGGGAACTGCATTCACTG	NM_001012406.1	[23]	125
Pax7	AGATCGCAGCAGGGGTAAAG	GACCCCACCAAGCTGATTGA	XM_021095458.1	Primerblast	209
Myl1	CTCTCAAGATCAAGCACTGCG	GCAGACACTTGGTTTGTGTGG	NM_214374.2	[24]	198
Myf5	GCTGCTGAGGGAACAGGTGGA	CTGCTGTTCTTTCGGGACCAGAC	NM_001278775.1	[25]	135
MSTN	CCCGTCAAGACTCCTACAACA	CACATCAATGCTCTGCCAA	NM_005259.3	[25]	141
ACT	CGGGCAGGTCATCACCATC	CGTGTTGGCGTAGAGGTCCTT	XM_005670976.2 ^##^	[25]	160
MYH1	CCAGGGAGAGATGGAGGACA	TCAAGTTCACGTACCCTGGC	NM_001104951.2	Primerblast	258
Des	ACACCTCAAGGATGAGATGGC	CAGGGCTTGTTTCTCGGAAG	NM_001001535.1	[24]	176
Myf6	AGTGGCCAAGTGTTTCGGATC	CGCGAGTTATTTCTCCCCCA	NM_001244672.1	Primerblast	179
ACTA1	ACCCGACGCCATGTGTGA	GTCGCCCACGTAGGAATCTT	NM_001167795.1	Primerblast	184
MyoD1	CACTACAGCGGTGACTCAGACGCA	GACCGGGGTCGCTGGGCGCCTCGCT	NM_001002824.1	[25]	145

**Table 2:** Results of one way ANOVA performed on log-transformed values indicating significant results for all genes.

Gene	*p*-Value
Pax7	0.001
MyoD1	<0.001
Myf5	0.005
MyoG	0.017
Myf6	0.019
Des	0.005

**Table 3:** Results of Kruskal–Wallis tests indicate significant results. However, Pax7 and Myh1 are not significant.

Gene	*p*-Value
Pax7	0.184
Myh1	0.158
MyoD1	0.006
Myf5	0.028
MyoG	0.017
Myf6	0.045
Des	0.008

## Data Availability

The data generated and explored are included in the manuscript. The raw data of the presented information are available to academic colleagues for non-commercial purposes upon their justified request.
